# Supplementary material for: Effects of soil pH on the growth, soil nutrient composition, and rhizosphere microbiome of Ageratina adenophora
Source: PeerJ. 2024 Apr 16;12:e17231. doi: 10.7717/peerj.17231 (PMC11027909; doi:10.7717/peerj.17231)
Supplement: Supplemental Information 2 [file peerj-12-17231-s002.docx]

| Phylum name | Bulk soil/pH 6.5a | Bulk soil/pH 9.0a | Bulk soil/pH 6.5b | Bulk soil/pH 5.5b | Bulk soil/pH 7.2b | Bulk soil/pH 9.0b | pH 6.5a/pH 6.5b | pH 6.5a/pH 5.5a | pH 6.5a/pH 9.0a | pH 5.5a/pH 9.0a | pH 5.5a/pH 5.5b | pH 7.2a/pH 9.0a | pH 7.2a/pH 7.2b | pH 6.5b/pH 5.5b | pH 5.5b/pH 7.2b | pH 5.5b/pH 9.0b |
| --- | --- | --- | --- | --- | --- | --- | --- | --- | --- | --- | --- | --- | --- | --- | --- | --- |
| Ascomycota | − | − | 0.001 | − | 0.006 | 0.021 | 0.014 | − | − | − | 0.024 | − | 0.005 | 0.035 | − | − |
| Basidiomycota | 0.005 | 0.002 | − | − | − | 0.024 | − | 0.007 | − | 0.002 | − | − | − | − | − | − |
| Chytridiomycota | − | 0.0001 | 0.002 | − | 0.006 | 0.014 | − | − | 0.031 | 0.004 | − | 0.007 | − | − | − | − |
| Mortierellomycota | 0.021 | 0.001 | 0.001 | − | 0.016 | − | − | − | − | 0.009 | − | 0.018 | − | − | − | − |
| Glomeromycota | − | − | 0.019 | 0.002 | 0.0001 | 0.017 | − | − | − | − | 0.018 | − | 0.006 | − | − | − |
| Rozellomycota | − | 0.045 | 0.027 | − | − | 0.003 | − | − | − | − | − | − | − | 0.005 | 0.048 | 0.0001 |
| Olpidiomycota | − | − | − | − | − | − | − | − | − | − | − | − | − | − | − | − |
