# Supplementary material for: Effects of soil pH on the growth, soil nutrient composition, and rhizosphere microbiome of Ageratina adenophora
Source: PeerJ. 2024 Apr 16;12:e17231. doi: 10.7717/peerj.17231 (PMC11027909; doi:10.7717/peerj.17231)
Supplement: Supplemental Information 3 [file peerj-12-17231-s003.docx]

| Genus name | Bulk soil/pH 6.5a | Bulk soil/pH 5.5a | Bulk soil/pH 7.2a | Bulk soil/pH 9.0a | Bulk soil/pH 6.5b | Bulk soil/pH 5.5b | Bulk soil/pH 7.2b | Bulk soil/pH 9.0b | pH 6.5a/pH 6.5b | pH 5.5a/pH 5.5b | pH 7.2a/pH 7.2b | pH 9.0a/pH 9.0b | pH 6.5a/pH 5.5a | pH 6.5a/pH 7.2a | pH 6.5a/pH 9.0a | pH 5.5a/pH 7.2a | pH 5.5a/pH 9.0a | pH 7.2a/pH 9.0a | pH 6.5b/pH 5.5b | pH 6.5b/pH 7.2b | pH 6.5b/pH 9.0b | pH 5.5b/pH 7.2b | pH 5.5b/pH 9.0b | pH 7.2b/pH 9.0b |
| --- | --- | --- | --- | --- | --- | --- | --- | --- | --- | --- | --- | --- | --- | --- | --- | --- | --- | --- | --- | --- | --- | --- | --- | --- |
| norank Micropepsaceae | − | 0.009 | − | − | − | 0.031 | − | − | − | − | − | − | 0.045 | − | − | − | 0.0001 | 0.01 | − | − | − | − | 0.003 | − |
| Massilia | 0.035 | − | 0.027 | 0.001 | 0.018 | − | − | 0.0001 | − | − | − | − | − | − | − | − | 0.021 | − | − | − | − | − | 0.002 | 0.04 |
| Rhodanobacter | − | 0.001 | − | − | − | 0.005 | 0.024 | − | − | − | − | − | − | − | − | − | 0.005 | − | − | − | − | − | 0.001 | 0.006 |
| unclassified Comamonadaceae | − | − | − | − | 0.024 | 0.002 | 0.027 | 0.0001 | − | − | − | 0.045 | − | − | − | − | − | − | − | − | − | − | − | − |
| Bryobacter | − | − | − | 0.016 | − | − | − | 0.045 | − | − | − | − | 0.035 | − | − | − | 0.002 | − | 0.04 | − | − | − | 0.004 | 0.01 |
| norank Vicinamibacterales | − | − | − | − | − | − | − | 0.021 | 0.018 | 0.021 | 0.024 | − | − | − | − | − | − | − | − | − | − | − | − | − |
| Granulicella | 0.01 | 0.006 | 0.024 | − | − | − | − | − | − | − | − | − | − | − | 0.012 | − | 0.007 | 0.027 | − | − | − | − | 0.014 | − |
| unclassified Micrococcaceae | − | − | − | 0.035 | − | − | − | − | − | − | − | − | − | − | − | − | 0.003 | − | − | 0.045 | − | 0.012 | 0.006 | − |
| Gemmatimonas | − | − | − | − | 0.002 | 0.01 | 0.001 | − | 0.002 | − | 0.014 | − | − | − | − | − | − | − | − | − | − | − | − | − |
| Streptomyces | − | − | − | − | 0.001 | − | 0.022 | 0.002 | 0.008 | − | − | − | − | − | − | − | − | − | − | − | − | − | − | − |
| Phenylobacterium | − | − | − | 0.002 | 0.0001 | 0.003 | 0.031 | − | 0.019 | − | − | − | − | − | − | − | − | − | − | − | 0.016 | − | − | − |
| Burkholderia-Caballeronia-Paraburkholderia | − | − | − | 0.009 | 0.0001 | − | 0.018 | 0.001 | − | − | − | − | − | − | − | − | 0.031 | − | − | − | − | − | − | − |
| Pseudolabrys | − | − | − | 0.001 | − | − | 0.005 | 0.0001 | − | − | 0.031 | − | − | − | − | − | − | 0.001 | − | − | − | − | − | − |
| Edaphobacter | − | − | − | − | − | − | − | 0.045 | − | − | 0.027 | − | − | − | − | − | 0.021 | 0.007 | 0.045 | − | − | − | 0.014 | − |
| Reyranella | − | − | − | 0.001 | − | − | 0.045 | 0.007 | − | − | 0.014 | − | − | − | − | − | 0.005 | 0.0001 | − | − | − | − | − | − |
| norank Chitinophagaceae | 0.01 | 0.003 | 0.0001 | − | − | 0.018 | − | − | − | − | − | − | − | − | − | − | − | 0.012 | − | − | − | − | 0.035 | − |
| norank Vicinamibacteraceae | − | − | − | − | 0.021 | − | − | 0.031 | 0.001 | 0.04 | − | − | − | − | − | − | − | − | − | − | − | − | − | − |
| Dongia | − | − | − | 0.004 | − | − | − | 0.002 | − | − | 0.016 | − | − | 0.016 | − | − | − | 0.001 | − | − | − | − | 0.007 | − |
| norank Caulobacteraceae | − | 0.004 | 0.016 | − | − | 0.003 | − | − | − | − | − | − | − | − | − | − | 0.027 | − | − | − | − | − | 0.001 | 0.045 |
| Acidibacter | − | − | − | − | 0.04 | − | − | − | − | − | 0.027 | − | − | − | 0.004 | − | − | 0.005 | − | 0.003 | − | − | − | − |
| Chujaibacter | − | − | − | − | − | − | − | − | 0.01 | − | − | − | − | − | 0.035 | − | − | 0.024 | 0.012 | − | − | − | 0.003 | 0.027 |
| Mucilaginibacter | − | 0.009 | − | − | − | − | − | − | − | − | − | − | − | − | 0.027 | − | 0.001 | 0.04 | − | − | − | − | 0.003 | 0.045 |
| Novosphingobium | − | − | − | 0.003 | 0.004 | − | − | − | − | − | − | − | − | − | 0.045 | 0.016 | 0.0001 | − | − | 0.035 | − | − | − | − |
| Devosia | − | − | − | 0.016 | − | − | − | − | − | − | − | − | − | − | 0.016 | − | − | 0.01 | − | 0.014 | − | − | − | − |
| unclassified Sphingomonadaceae | − | − | 0.003 | 0.002 | − | 0.002 | − | − | − | − | − | 0.009 | − | − | − | − | − | − | − | − | − | − | 0.009 | − |
| Defluviitoga | − | − | 0.001 | 0.001 | − | − | 0.042 | 0.019 | − | − | − | − | − | − | − | 0.031 | 0.035 | − | − | − | − | − | − | − |
| Acidipila | − | − | − | 0.016 | 0.001 | − | − | 0.003 | − | − | − | − | − | − | − | − | 0.007 | − | 0.018 | − | − | − | 0.045 | − |
| unclassified Xanthobacteraceae | − | 0.001 | − | − | − | − | 0.016 | 0.001 | − | 0.033 | − | 0.021 | − | − | − | − | 0.021 | − | − | − | 0.033 | − | 0.033 | − |
| Arenimonas | − | − | − | 0.045 | 0.001 | − | 0.01 | 0.005 | 0.005 | − | − | − | − | − | − | − | 0.031 | − | − | − | − | − | − | − |
| unclassified Caulobacteraceae | − | − | − | − | 0.001 | 0.005 | 0.0001 | − | − | − | − | − | − | − | − | − | − | − | − | − | − | − | − | − |
| Flavobacterium | 0.002 | − | 0.012 | 0.0001 | 0.007 | − | − | − | − | − | − | 0.045 | 0.008 | − | − | 0.04 | 0.001 | − | − | − | − | − | − | − |
| unclassified Xanthomonadaceae | 0.024 | − | 0.007 | − | 0.001 | − | 0.0001 | − | − | − | − | − | − | − | − | − | − | − | − | − | 0.004 | − | − | 0.001 |
| unclassified Rhodanobacteraceae | − | − | − | − | − | − | − | 0.016 | 0.025 | − | − | − | − | − | 0.003 | − | 0.021 | − | − | 0.029 | − | 0.029 | − | 0.001 |
| Dokdonella | − | 0.035 | − | 0.005 | − | − | − | 0.001 | − | − | 0.031 | − | − | − | − | − | − | − | − | − | 0.016 | − | 0.005 | 0.0001 |
| norank Xanthobacteraceae | − | 0.001 | − | 0.009 | − | − | − | 0.001 | − | − | − | − | 0.024 | − | − | − | − | − | − | − | − | − | 0.045 | 0.012 |
| unclassified Oxalobacteraceae | 0.001 | 0.007 | 0.006 | 0.025 | − | − | − | 0.009 | 0.045 | 0.024 | − | − | − | − | − | − | − | − | − | − | − | − | 0.027 | − |
| norank Acetobacteraceae | − | − | − | − | − | − | − | − | 0.029 | − | − | − | − | − | 0.045 | − | 0.009 | − | 0.009 | − | − | − | 0.003 | − |
| Luteimonas | − | − | 0.005 | − | 0.001 | 0.035 | 0.0001 | − | − | − | − | − | − | − | − | − | − | − | − | − | 0.005 | − | − | 0.004 |
| Ellin6067 | − | − | − | − | 0.0001 | 0.024 | 0.005 | 0.001 | 0.018 | − | − | − | − | − | − | − | − | − | − | − | − | − | − | − |
| Dyella | − | − | − | − | − | − | − | − | 0.003 | − | − | − | − | − | 0.029 | − | 0.029 | 0.004 | − | 0.014 | − | − | − | − |
| Chitinophaga | − | − | − | 0.006 | − | 0.033 | 0.004 | 0.0001 | − | − | 0.005 | − | − | − | 0.045 | − | − | 0.008 | − | − | 0.021 | − | − | − |
| Pedobacter | 0.002 | − | 0.018 | 0.0001 | 0.004 | − | − | − | − | − | − | − | − | − | − | − | 0.012 | − | 0.016 | − | − | − | − | − |
| Sphingomonas | − | − | 0.002 | 0.002 | 0.0001 | − | − | − | − | − | − | − | − | − | − | 0.024 | 0.027 | − | 0.021 | 0.045 | − | − | − | − |
| unclassified Acetobacteraceae | − | − | − | 0.002 | 0.001 | − | 0.021 | 0.0001 | 0.031 | − | − | − | − | − | 0.045 | − | − | 0.029 | − | − | − | − | − | − |
| Stenotrophomonas | − | − | − | 0.001 | 0.04 | − | 0.016 | 0.003 | − | − | − | − | − | − | 0.045 | − | 0.009 | 0.006 | − | − | − | 0.029 | 0.007 | − |
| Adhaeribacter | 0.044 | − | − | 0.002 | 0.026 | − | − | 0.003 | − | − | − | − | 0.022 | − | − | − | 0.001 | − | − | − | − | − | 0.008 | 0.044 |
| norank WD2101-soil-group | − | − | − | 0.031 | 0.001 | 0.012 | 0.001 | − | 0.045 | − | 0.024 | − | − | − | − | − | 0.04 | − | − | − | 0.035 | − | − | − |
| Bordetella | − | − | − | 0.035 | − | − | 0.025 | 0.005 | − | − | 0.013 | − | − | − | − | − | 0.003 | 0.018 | − | − | − | − | 0.048 | − |
| Brevundimonas | − | − | 0.035 | 0.0001 | 0.005 | − | − | 0.001 | − | − | − | − | − | − | − | − | 0.001 | − | − | − | − | − | 0.021 | − |
| Ramlibacter | − | − | 0.031 | 0.002 | − | − | − | 0.0001 | − | − | − | − | − | − | − | − | − | − | − | − | 0.025 | − | 0.007 | − |
