# Supplementary material for: Effects of soil pH on the growth, soil nutrient composition, and rhizosphere microbiome of Ageratina adenophora
Source: PeerJ. 2024 Apr 16;12:e17231. doi: 10.7717/peerj.17231 (PMC11027909; doi:10.7717/peerj.17231)
Supplement: Supplemental Information 4 [file peerj-12-17231-s004.docx]

| Genus name | Bulk soil/pH 6.5a | Bulk soil/pH 5.5a | Bulk soil/pH 7.2a | Bulk soil/pH 9.0a | Bulk soil/pH 6.5b | Bulk soil/pH 5.5b | Bulk soil/pH 7.2b | Bulk soil/pH 9.0b | pH 6.5a/pH 6.5b | pH 6.5a/pH 5.5a | pH 6.5a/pH 7.2a | pH 6.5a/pH 9.0a | pH 5.5a/pH 7.2a | pH 5.5a/pH 9.0a | pH 5.5a/pH 5.5b | pH 7.2a/pH 9.0a | pH 7.2a/pH 7.2b | pH 9.0a/pH 9.0b | pH 6.5b/pH 5.5b | pH 6.5b/pH 7.2b | pH 6.5b/pH 9.0b | pH 5.5b/pH 7.2b | pH 5.5b/pH 9.0b | pH 7.2b/pH 9.0b |
| --- | --- | --- | --- | --- | --- | --- | --- | --- | --- | --- | --- | --- | --- | --- | --- | --- | --- | --- | --- | --- | --- | --- | --- | --- |
| Latorua | 0.002 | − | − | 0.002 | 0.001 | − | 0.035 | 0.045 | − | 0.04 | − | − | − | 0.04 | − | − | − | − | 0.009 | − | − | − | − | − |
| Mortierella | 0.021 | ⎼ | − | 0.001 | 0.001 | − | 0.016 | − | − | − | − | − | − | 0.009 | − | 0.018 | − | − | − | − | − | − | − | − |
| Leucocoprinus | 0.04 | ⎼ | − | 0.001 | 0.003 | − | 0.031 | 0.001 | − | − | − | − | − | 0.005 | − | − | − | − | − | − | − | − | 0.024 | − |
| Arthrographis | ⎼ | ⎼ | − | 0.002 | 0.045 | − | 0.014 | 0.001 | − | − | − | 0.021 | − | 0.009 | − | 0.031 | − | − | − | − | − | − | 0.021 | − |
| Aspergillus | ⎼ | ⎼ | − | − | − | − | − | − | − | − | − | − | − | − | − | − | − | − | − | − | − | − | − | − |
| Arachnomyces | ⎼ | ⎼ | 0.009 | 0.001 | 0.011 | − | − | 0.0001 | − | − | − | 0.037 | 0.047 | 0.006 | − | − | − | − | − | − | − | − | 0.01 | − |
| Chrysosporium | ⎼ | 0.005 | 0.002 | 0.002 | − | − | − | − | − | − | − | − | − | − | 0.035 | − | 0.021 | − | − | − | − | − | − | − |
| unclassified Spizellomycetaceae | − | − | − | 0.006 | 0.006 | 0.014 | 0.001 | 0.021 | − | − | − | − | − | − | − | 0.035 | 0.01 | − | − | − | − | − | − | − |
| Penicillium | − | − | − | − | 0.016 | − | − | 0.045 | 0.045 | − | − | − | − | 0.035 | − | 0.01 | 0.005 | − | 0.04 | − | − | − | − | − |
| unclassified Chytridiomycota | − | − | − | 0.003 | 0.0001 | − | 0.004 | 0.01 | 0.04 | − | − | − | − | 0.014 | − | − | − | − | 0.12 | − | − | − | − | − |
| Geomyces | 0.0001 | 0.008 | 0.001 | 0.013 | − | − | − | − | 0.014 | − | − | − | − | − | − | − | 0.042 | − | − | − | − | − | − | − |
| Pseudogymnoascus | 0.006 | 0.045 | 0.0001 | 0.001 | − | − | − | − | − | − | − | − | − | − | − | − | 0.04 | − | − | − | − | − | − | − |
| unclassified Sordariales | ⎼ | 0.002 | 0.024 | − | − | − | − | 0.016 | − | 0.007 | − | − | − | − | 0.045 | − | − | − | − | − | 0.045 | − | − | − |
| Archaeospora | − | − | − | − | − | 0.006 | 0.001 | 0.028 | − | − | − | − | − | − | − | − | 0.005 | − | − | − | − | − | − | − |
| Sagenomella | − | − | − | − | − | − | − | − | − | − | − | 0.024 | − | 0.006 | − | 0.003 | 0.005 | − | − | − | 0.045 | − | − | − |
| Conocybe | ⎼ | 0.04 | − | − | 0.001 | 0.003 | − | − | 0.035 | − | − | − | − | 0.035 | − | − | − | − | − | − | − | − | − | − |
| Clitopilus | − | − | − | − | − | − | − | − | − | − | − | − | − | − | − | − | − | − | − | − | − | − | − | − |
| Arcopilus | − | − | − | 0.024 | − | − | − | 0.003 | 0.014 | − | − | − | − | − | − | − | − | − | − | 0.027 | 0.0001 | − | 0.04 | − |
| Talaromyces | 0.024 | − | − | 0.014 | 0.003 | − | 0.012 | 0.001 | − | − | − | − | − | − | − | − | − | − | − | − | − | − | − | − |
| Cladosporium | − | − | − | − | − | − | − | − | 0.024 | − | − | − | − | − | − | − | 0.027 | 0.01 | − | − | − | − | − | − |
| unclassified Rozellomycota | − | − | − | − | 0.033 | − | − | 0.004 | − | − | − | − | − | − | − | − | − | − | 0.006 | − | − | − | 0.0001 | − |
| unclassified Basidiomycota | − | − | − | − | 0.0001 | 0.016 | 0.005 | 0.003 | 0.016 | − | − | − | − | − | − | − | 0.027 | − | − | − | − | − | − | − |
| Apiotrichum | − | − | − | − | − | − | − | − | − | − | − | − | − | − | − | − | − | − | − | − | − | − | − | − |
| Rhizophlyctis | 0.024 | − | − | 0.001 | − | − | − | 0.018 | − | − | − | − | − | 0.003 | − | 0.036 | − | − | − | − | − | − | − | − |
| unclassified Chaetomiaceae | − | 0.01 | 0.024 | − | − | − | − | − | 0.027 | − | − | − | − | − | − | − | 0.021 | − | 0.04 | − | − | − | − | − |
| unclassified Hypocreales | − | − | − | − | 0.021 | − | − | 0.037 | 0.005 | − | − | − | − | − | − | − | − | − | 0.031 | − | − | − | − | − |
| unclassified Powellomycetaceae | 0.029 | − | − | 0.001 | 0.023 | − | 0.029 | 0.019 | − | − | − | − | − | − | − | 0.003 | − | − | − | − | − | − | − | − |
| Trichocladium | − | − | − | 0.0001 | − | − | 0.027 | 0.003 | − | − | − | 0.012 | − | 0.021 | − | 0.031 | − | − | − | − | 0.045 | − | − | − |
| Dactylella | − | − | − | 0.007 | − | 0.0001 | − | 0.008 | − | − | − | 0.021 | − | − | − | − | − | − | 0.002 | 0.02 | − | − | − | − |
| Funneliformis | − | − | − | − | 0.002 | 0.046 | 0.009 | 0.001 | 0.013 | − | − | − | − | − | − | − | − | − | − | − | − | − | − | − |
| Saitozyma | − | 0.035 | − | − | − | − | − | − | 0.033 | − | − | − | − | 0.024 | − | − | 0.035 | − | 0.009 | − | − | − | − | 0.042 |
| unclassified Lasiosphaeriaceae | − | − | − | 0.005 | 0.013 | − | − | 0.039 | − | − | − | − | − | 0.009 | − | 0.009 | − | − | 0.003 | − | − | − | 0.011 | − |
| Phialemonium | − | − | 0.024 | 0.002 | − | − | 0.001 | 0.016 | − | − | − | − | − | − | − | − | − | − | − | 0.027 | − | 0.027 | − | − |
| unclassified Dipodascaceae | − | 0.009 | 0.006 | − | − | − | 0.005 | − | − | 0.04 | 0.031 | − | − | − | − | − | − | − | − | 0.004 | − | − | − | − |
| Trichoderma | − | − | − | − | 0.006 | − | − | − | − | 0.029 | − | − | − | − | − | − | − | − | 0.001 | − | − | 0.016 | 0.015 | − |
| Articulospora | ⎼ | 0.048 | 0.013 | 0.001 | − | 0.019 | 0.029 | − | − | − | − | − | − | − | − | − | − | 0.018 | − | − | − | − | − | − |
| Chaetomium | − | − | − | − | − | − | − | − | − | − | − | − | − | − | − | − | − | − | − | − | − | − | − | − |
| Schizothecium | 0.014 | − | − | 0.0001 | 0.027 | − | − | 0.006 | − | − | − | − | − | 0.005 | − | 0.016 | − | − | − | − | − | − | − | − |
| unclassified Pleosporales | − | − | − | − | 0.006 | 0.0001 | 0.01 | 0.009 | 0.04 | − | − | − | − | − | − | − | − | − | − | − | − | − | − | − |
| Gibberella | − | − | − | − | − | − | − | − | − | − | − | − | − | − | − | − | − | − | − | − | − | − | − | − |
| Lecanicillium | − | − | − | − | − | − | − | 0.029 | − | − | − | − | − | 0.035 | − | 0.045 | − | − | − | − | − | − | − | − |
| Cercophora | − | − | − | 0.013 | 0.006 | − | − | 0.001 | − | − | − | − | − | − | − | 0.013 | − | − | − | − | − | − | − | 0.01 |
| unclassified Tremellodendropsidales | − | − | − | − | − | 0.009 | − | 0.034 | − | − | − | − | − | 0.046 | − | − | − | − | 0.003 | 0.014 | − | − | 0.001 | 0.005 |
| Oidiodendron | ⎼ | 0.016 | 0.018 | − | − | − | − | − | − | 0.025 | 0.029 | − | − | 0.027 | − | 0.031 | 0.035 | − | − | − | − | − | − | − |
| Coniochaeta | − | − | − | 0.012 | − | − | − | − | 0.022 | − | − | − | − | − | − | − | − | 0.006 | − | − | − | − | − | − |
| Epicoccum | − | − | − | 0.002 | − | − | − | 0.007 | − | − | − | 0.021 | − | − | − | − | − | − | − | − | − | − | 0.006 | − |
| Acremonium | − | − | − | − | 0.017 | − | 0.033 | − | − | − | − | 0.033 | − | − | − | − | − | − | − | − | − | − | − | − |
| unclassified Ascomycota | − | 0.031 | − | − | − | − | − | − | − | − | − | − | − | − | − | − | 0.018 | − | 0.029 | − | − | − | − | − |
| Fusarium | − | − | − | 0.021 | 0.019 | − | − | − | − | − | − | − | − | 0.027 | − | 0.004 | 0.014 | − | − | − | − | − | − | − |
| Acrophialophora | − | − | − | 0.009 | 0.009 | − | 0.048 | 0.009 | − | − | − | − | − | 0.005 | − | 0.004 | 0.025 | − | − | − | − | − | − | − |
