# Supplementary figures and images for: Effects of soil pH on the growth, soil nutrient composition, and rhizosphere microbiome of Ageratina adenophora
Source: PeerJ. 2024 Apr 16;12:e17231. doi: 10.7717/peerj.17231 (PMC11027909; doi:10.7717/peerj.17231)

(A)

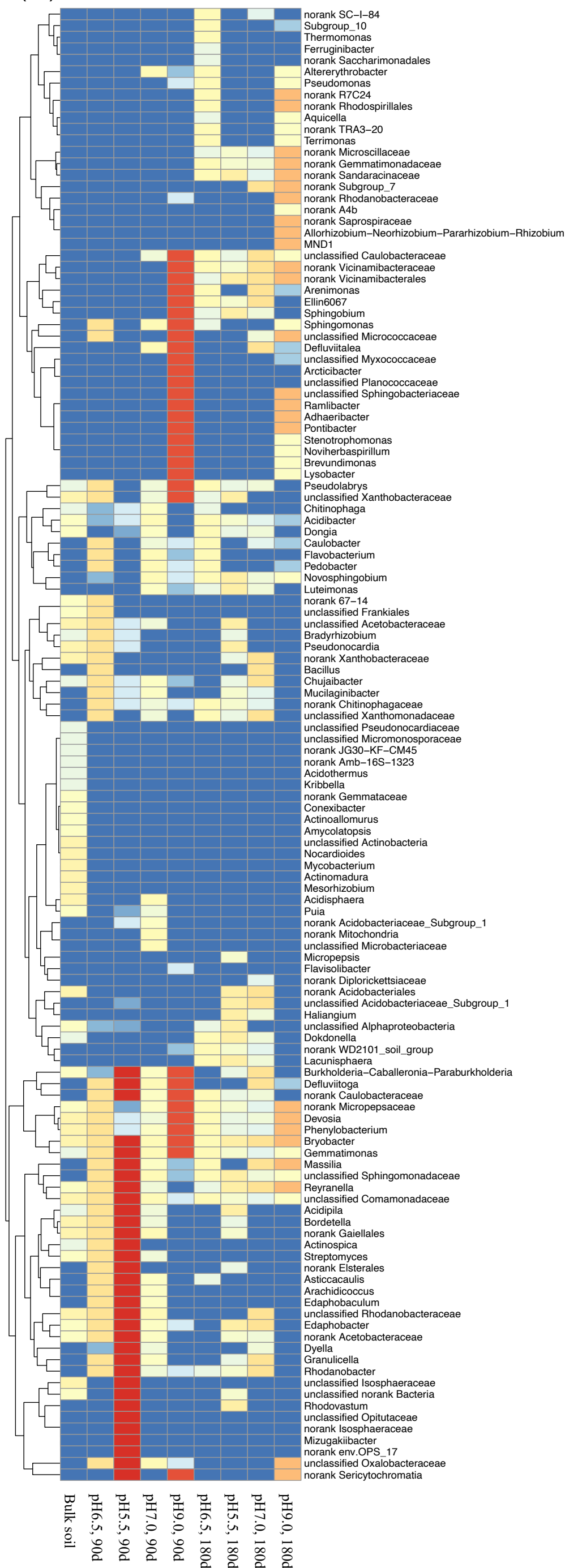

(B)

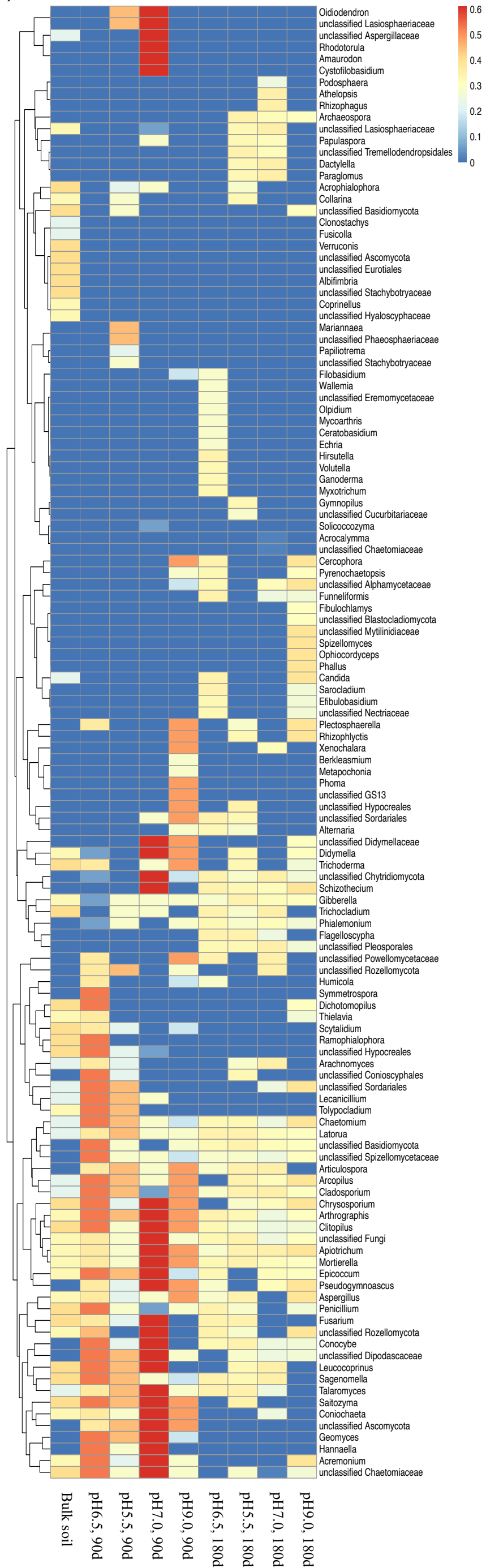

Supplement: Supplemental Information 5 [file peerj-12-17231-s005.pdf]
